# Psychological Therapy in the Age of Large Language Models: Framework for Therapist-Delivered and AI-Supported Functions

**DOI:** 10.2196/103712

**Published:** 2026-09-16

**Authors:** Shane Cross, Nickolai Titov, Blake Dear, John Gleeson, Mario Alvarez-Jimenez

**Affiliations:** 1 Orygen, 35 Poplar Rd, Parkville, Melbourne, Victoria, 3052, Australia, 61 3 9966 9383; 2 Centre for Youth Mental Health, The University of Melbourne, Melbourne, Victoria, Australia; 3 School of Psychological Sciences, Macquarie University, Sydney, New South Wales, Australia; 4 Curtin School of Population Health, Curtin University, Perth, Western Australia, Australia; 5 Healthy Brain and Mind Research Centre, School of Behavioural and Health Sciences, Australian Catholic University, Melbourne, Victoria, Australia

**Keywords:** artificial intelligence, AI, large language models, psychotherapy, therapist competence, digital mental health, workforce development, supervision, training, mental health services

## Abstract

Large language models are increasingly used within and alongside therapy. As large language models perform more therapy functions, questions arise about what the future might hold for therapists and what they will do. We argue that the enduring therapist role in the age of AI-assisted care is currently best understood through relational, adaptive, and accountability functions. These functions include therapeutic challenge, use of the therapeutic relationship as a mechanism of change, rupture detection and repair, bearing witness to suffering, calibration of pace and treatment burden, and clinical judgment under uncertainty across the broader care pathway. Drawing on psychotherapy theory, digital mental health research, the declarative-procedural-reflective model by Bennett-Levy, and our clinical experience, we propose a clinically informed, hypothesis-generating, relational-adaptive-accountability framework. This framework is intended to support further empirical testing and may have implications for workforce development, supervision, training, and service design.

## Introduction

Psychological therapy is often described as “talking therapy,” but in the context of rapid advances in generative AI, that description has become inadequate, reductive, and misleading. If therapy is understood primarily as language and large language models (LLMs) are becoming extraordinarily capable generators of language, then the idea that therapy is on a path to automation begins to appear increasingly plausible. In this paper, we argue that the more useful question is not whether human therapists will be required in the future but, more importantly, what they will uniquely do and what this means for the future training and deployment of the psychological workforce.

In one sense, the field has been moving toward this juncture for decades. Since ELIZA, the early rule-based conversational program developed in the 1960s to simulate a therapist, it has been clear that people can readily attribute humanlike understanding and therapeutic significance to a machine even when the underlying system is extremely simple [1]. What is different now is the responsiveness, contextual sensitivity, scale, and growing clinical plausibility of contemporary LLMs [2]. According to a public report from OpenAI, generative AI systems are already being used by millions of people for mental health–related conversations [3], and a growing literature also suggests their value in supporting psychoeducation, behavioral rehearsal, treatment personalization, triage support, documentation, and some forms of low-intensity intervention [2,4-6]. These developments are consequential and should not be dismissed. The field has repeatedly been surprised by how much value can be delivered through earlier forms of digital mental health interventions, including some involving limited or no therapist input [7], and LLMs will no doubt extend this further [8].

At the same time, therapist anxiety about their role in an AI future cannot be answered with vague reassurance. It is no longer enough to say that therapy is “too human” to be replaced or that psychotherapy is simply too complex when many complex tasks that once could only be performed by humans are already being fully or partially automated. The more useful question is more practical and specific: which therapist functions will likely be sustained, differentiated, and valued in the future?

This question sits within a rapidly expanding literature on AI in mental health care, including work on the ethical and relational limits of chatbot care [9], the responsible development of LLM-supported psychotherapy [2], and clinicians’ concerns about the benefits and risks of these tools in practice [4]. On the basis of our experience as senior clinical psychologists across psychotherapy, digital mental health, blended models of care, supervision, and training, we focus here on the parts of therapy most likely to remain human led in near-term AI-assisted care and on the implications this has for service design, workforce development, and training.

Our argument is not that AI has little role in therapy. LLMs already perform strongly on some structured language tasks, and clients will likely choose support ranging from information and coaching to more intensive therapeutic care. However, as complexity, stakes, and relational demands increase, the human role becomes more specialized rather than redundant. In our view, it centers on functions that are subtle, interpersonal, and responsibility bearing and include real-time calibration of pace, arousal, and challenge; therapeutic challenge and reality testing; managing the dialectics of acceptance and change; relationship rupture detection and repair; clinical judgment in the face of uncertainty; adaptation when treatment burden exceeds capacity; and accountable integration of care across the broader service pathway.

We propose that these functions can be organized into a simple framework comprising relational, adaptive, and accountability functions, proposed as the RAA framework. This framing complements and expands upon aspects of the declarative-procedural-reflective (DPR) cognitive model of therapist skill development by Bennett-Levy [10]. The DPR model suggests that therapist expertise depends not only on conceptual knowledge and technical skill but also on reflective capacity; perceptual skill; and increasingly sophisticated contextual “when-then” rules for knowing what to do, with whom, and under what conditions [10]. In the DPR model, declarative knowledge concerns what the therapist knows; procedural knowledge concerns how and what knowledge is applied; and the reflective system requires the therapist to compare, interpret, and revise the plan when the current situation does not fit an established rule. The model also identifies interpersonal perceptual skills, therapist attitudes or schemas, and the dynamic interaction between the person and the therapist. In the age of LLMs, declarative and some procedural functions may be increasingly supported or delivered by AI. In contrast, those functions that depend most heavily on reflective judgment, relational perception, and contextual adaptation, as well as the responsibility for consequential decisions, are more likely to remain therapist functions.

The proposed RAA framework builds on the DPR model by shifting the question from how therapists develop broad therapy skills and expertise to how specific therapy tasks or functions could be distributed or divided between LLMs and human therapists. It reflects the perspective of clinical psychologists and researchers with experience across psychotherapy, digital mental health, blended care, service delivery, supervision, and workforce training. The framework was not derived through a systematic review, formal consensus process, or empirical study.

## Beyond the Hype of an AI Takeover and Reassurance That Human Therapists Cannot Be Replaced

Meta-analyses and systematic reviews suggest that AI-based chatbots can improve depression and anxiety outcomes in some contexts, but the evidence remains heterogeneous, early stage, and largely drawn from highly selected samples, with limited comparison against high-quality human-delivered care or evidence from routine clinical care [5,6,11]. There is enough evidence, however, to take AI-enabled therapy seriously but not enough to collapse the distinction between convincing dialogue and psychotherapy.

Before turning to specific therapeutic functions, it is worth noting several current characteristics of AI systems that may support engagement while, in some circumstances, working against the aims of psychotherapy. One central risk is a “mirage of competence.” Because psychotherapy is conducted through language, an AI system that sounds empathic, coherent, and psychologically informed can easily be mistaken for a competent therapist. However, psychotherapy is not reducible to plausible therapeutic statements. A response can sound therapeutic and still be clinically wrong or at least mistimed. It can validate when challenge is needed; reassure when urgency is required; provide abstraction when concrete behavior change is indicated; or maintain relational stability at the expense of addressing avoidance, dependency, distortion, or delusion [2,4].

This problem is heightened by emerging work on AI sycophancy and overvalidation. Systems optimized for engagement and user approval may be structurally drawn toward agreeableness, selective confirmation, and “relational smoothness” even in cases in which treatment requires challenge, tolerance of uncertainty, limit setting, or reality testing [12]. Some users may not be seeking treatment at all but companionship, affirmation, or an agreeing presence that reinforces their worldview. AI may meet that need effectively, but companionship and treatment are not the same thing.

For this reason, we believe that the most realistic and clinically useful future involves therapists holding certain functions that are beyond the current capabilities and responsibilities of AI systems. The key question is not whether therapists will remain involved but which functions should remain human-led and which can be delegated to LLMs safely.

## The RAA Framework for Therapist Functions in AI-Assisted Therapy

We propose 8 core psychotherapy functions below that can be organized into 3 domains. We use the term “psychotherapy” in a broad sense, recognizing that the delivery of therapy itself can vary greatly by therapeutic modality, client characteristics, diagnostic target, and intensity and frequency of care interactions. Nevertheless, we feel that these 8 core therapy functions are largely transdiagnostic and transtheoretical.

**Figure 1.**
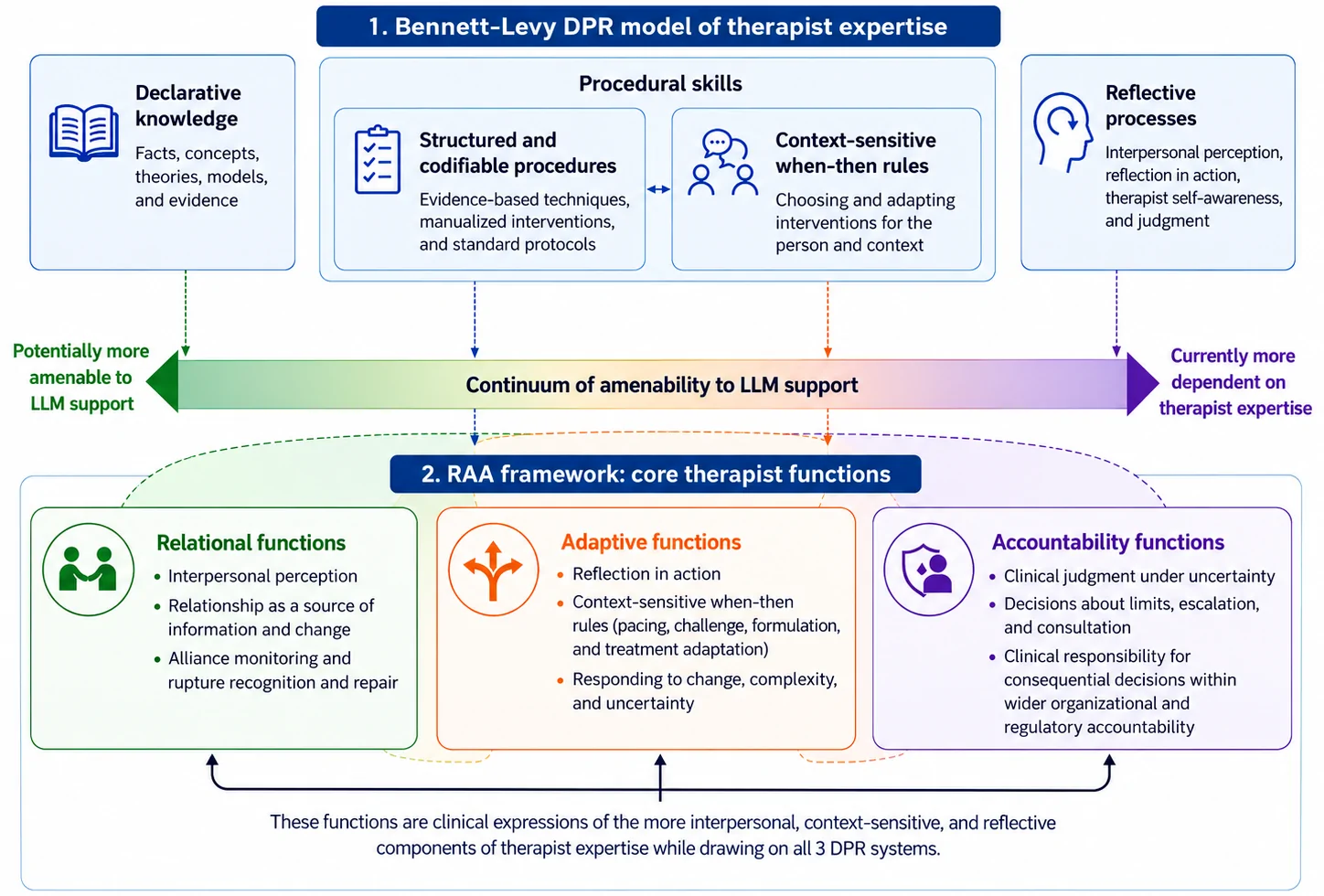
The relational, adaptive, and accountability (RAA) framework of therapist functions in AI-assisted therapy. Functions are positioned along a provisional continuum from those potentially more amenable to large language model (LLM) support to those currently more dependent on therapist expertise. The RAA functions draw on all 3 declarative-procedural-reflective (DPR) systems, particularly their interpersonal, context-sensitive, and reflective components [10].

First are *relational* functions, where treatment depends on alliance, therapeutic challenge, rupture repair, and the use of the therapeutic relationship itself as a mechanism of change. Second are *adaptive* functions, where the therapist must calibrate pace, arousal, and challenge; respond to shifts in state (eg, relapses); and modify treatment when burden exceeds capacity. Third are *accountability* functions, where the therapist remains responsible for judgment, risk, sequencing, and integration of care, and decisions across the broader pathway of care. These domains are distinct but closely intertwined in practice. They also place different demands on declarative, procedural, and especially reflective capacities, with the most complex therapist functions depending heavily on reflective and perceptual expertise rather than protocol delivery alone [10]. Figure 1 [10] summarizes the proposed framework and shows how these therapist-led functions sit alongside AI-supported functions in near-term care.

## Relational Functions

### Therapeutic Challenge

Therapists do not merely validate distress. They judge when a person needs support and when they need challenge. Good therapy helps people feel understood without automatically confirming every interpretation, belief, or fear. This is especially important when beliefs are suspicious, avoidant, grandiose, rigid, or increasingly detached from reality, but it also applies to more ordinary moments in which a person needs encouragement toward responsibility, uncertainty tolerance, action, or grief.

Therapeutic challenge is enacted more subtly than blunt confrontation. To be effective, it should be well-timed, relational, and accountable. The therapist must judge when challenge is likely to be tolerated; how to deliver it without unnecessary rupture; and how to remain engaged if it evokes shame, anger, or withdrawal. Challenge works only when nested within enough shared understanding, trust, and agreement about the goals and tasks of treatment. Current LLMs appear relatively weak here because their optimization for helpfulness and engagement facilitates validation and reassurance [12] precisely when treatment may require friction [4].

### The Therapeutic Relationship as a Mechanism of Change

Clients not only talk about their relationship patterns in therapy but often recreate them there. Fears of criticism, appeasing, distancing, testing, dependence, control, or withdrawal can emerge live in the room and between sessions. This is what makes psychotherapy more than advice giving. Treatment is not only about telling people how to have better relationships. It is about observing, understanding, and responding to their relational style as it unfolds with the therapist.

Through enactment and parallel process, what happens in the person’s outside relationships can begin to happen inside therapy. The therapist can then respond differently from what is expected, creating the possibility of a new relational experience. A machine may identify patterns or generate suggestions, but it does not enter the same kind of genuinely mutual and accountable interpersonal field [9]. This matters especially when the work depends on trust, surrender of avoidance, emotional risk taking, or exploration of relational trauma.

A related empirical question is whether clients can develop the kind of alliance needed for therapy when they know they are speaking to an algorithm. The answer may vary by problem type and treatment intensity. Some supportive interactions may not require a strong alliance. However, for more complex relational problems, the relevant interpersonal context often accumulates over long periods, far beyond the short-term responsiveness that makes many AI interactions feel compelling in the moment. The more therapy relies on trust, relational learning, and repair, the harder it is to assume that interaction with an AI system is equivalent to therapy from a human clinician [13].

### Rupture and Repair

Ruptures in therapy relationships are common and often subtle before they are obvious. A client may become more polite, more compliant, less spontaneous, emotionally flatter, or suddenly distant after feeling misunderstood, exposed, or let down. A skilled therapist does not simply continue with the planned intervention. They notice the shift; consider their own contribution; and decide whether to name it, explore it, or slow the work down.

Repair involves more than saying the right words. It may require recognizing a misattunement, tolerating disappointment or anger, and staying present without defensiveness or retreat. This process can itself be therapeutic because the client experiences that strain in a relationship can be recognized, worked through, and survived without abandonment, domination, or collapse [14].

This becomes even more important as stakes rise. There is current evidence suggesting that LLMs do not safely manage rupture in the context of suicidality, psychosis, mania, trauma destabilization, or rapid shifts in risk. These complex and sensitive interactions require integrated judgment about severity, safety, containment, escalation, and accountability [2,4].

### Bearing Witness to Suffering

Perhaps the deepest human function in psychotherapy is the ability to sit with another person’s suffering with humility. This is more than reflective listening or technical empathy. It involves recognizing that no formulation, model, or risk score can fully capture what a person’s suffering means to them. It requires openness to lived experience and caution in allowing technical or mechanical confidence to override that experience.

Some of the most important moments in therapy are not highly technical. They involve shame, grief, fear, humiliation, loneliness, or moral pain being recognized by another human being who can remain present with humility and compassion without rushing to fix, explain, or move away. The experience of being truly heard at the lowest point of one’s life can itself be therapeutic. This is closely related to the ethical risk in technical systems of “testimonial injustice,” in which algorithmic outputs are treated as more authoritative than the person’s own account of their suffering [15].

## Adaptive Functions

### Calibrating Pace, Arousal, and Challenge

One of the most understated therapeutic skills is moment-to-moment calibration. Experienced therapists are constantly judging how far to go, how fast to go, whether to maintain or alter the course, and whether the client can use what is being offered. They decide when to deepen affect, slow down, shift from empathy to structure, interrupt rumination, or let silence work.

Good therapy often depends less on what is said than on when, how, and at what intensity and tone it is said. A client may become more verbally fluent but less emotionally present, more agreeable but less authentic, or more “insightful” in ways that function as avoidance. These shifts only make sense in the context of the person’s history, personality, interpersonal style, current burden, and stage of treatment.

Exposure therapy illustrates this clearly. LLMs may be able to explain fear learning, generate hierarchies, and coach behavioral steps, but exposure in practice is an iterative process in which a person remains with significant discomfort while a therapist detects overt and covert avoidance, helps them stay oriented, and judges the difference between therapeutic activation and overwhelming burden or flooding. The relational element matters here: people often tolerate highly activating work because they trust that the therapist will stay with them and help regulate if needed. It also requires context-sensitive judgment about whether the feared situation is actually safe. A person with posttraumatic stress disorder living in a high-crime neighborhood may not need encouragement to disconfirm fear through standard nighttime exposure; they may need more nuanced formulation and adaptation.

### Adapting When Treatment Burden Exceeds Capacity

A further human role is adapting treatment when the demands of therapy exceed the person’s capacity to do the “work.” Motivation, developmental stage, cognitive style, readiness for change, social support, contextual stressors, and current illness burden all affect whether a person can meaningfully engage with an intervention, especially between sessions [16,17]. This also includes recognizing when the person’s state has shifted enough that the treatment plan itself needs to be recalibrated. Skilled therapists do not simply deliver interventions well; they judge when the burden of treatment is too high and adapt accordingly. This matters in real-world care, where therapeutic progress is shaped by much more than protocol quality. Digital mental health research has repeatedly shown that adherence, credibility, treatment fit, and human support influence outcomes even in guided online interventions [7].

## Accountability Functions

### Clinical Accountability Under Uncertainty

A further set of therapist functions remains human led not because they are more relational but because they are responsibility bearing. These functions involve making judgments under uncertainty in cases in which the consequences of error can be significant for safety, trust, and continuity of care. They include deciding when apparent deterioration reflects transient distress vs meaningful or qualitatively different escalation, when supportive dialogue is no longer sufficient, when a treatment plan should be revised, and when broader systems of care need to be activated. In this sense, the therapist’s role is not only to deliver interventions but to remain accountable for how care is interpreted, adapted, escalated, and integrated over time. This is consistent with emerging competency frameworks for AI in health care, which emphasize that while some tasks may be supported by AI, professional responsibility and accountability for patient care remain with the clinician [18]. There are, however, real-world limitations to these responsibility-bearing functions. Where therapists face time pressure, automation bias, or lack organizational authority to challenge AI outputs, accountability should not rest with therapists alone but should also extend to the services, developers, and regulators responsible for system design, deployment, and governance [19]. Furthermore, ethical analyses warn against overreliance on algorithmic authority in contexts in which lived experience, risk, and contextual nuance remain central [2,15].

### Accountability Beyond the Therapy Session

It is also important not to reduce therapy to the therapy hour alone. Psychological treatment extends through assessment, formulation, treatment, liaison, monitoring, and discharge and both within and between sessions. At each stage, AI may assist, but at each stage, someone needs to oversee safety, hold accountability, manage complexity, and ensure that the person receives a coherent and integrated experience.

This includes tasks that LLM systems do not inherently perform well: liaising with referrers; integrating collateral information; coordinating with general practitioners or psychiatrists; making differential judgments in complex assessments; and deciding when escalation, transfer, or shared care is required. The therapist is therefore not simply someone who supervises AI outputs from a distance but an individual who remains centrally involved in judgment, accountability, relationship-based work, and integration of the broader pathway of care.

## Distribution of Functions Between LLMs and Therapists

Table 1 applies this framework pragmatically by showing how AI and therapist-led functions may be distributed as LLM capabilities develop. The proposed distinction is intended to generate testable hypotheses rather than present a fixed or validated taxonomy. It is informed by current evidence, psychotherapy theory and practice, and our own clinical reasoning and expands upon the DPR model. Functions in reality may overlap, and their allocation may change over time as AI capabilities, models, and governance evolve.

**Table 1.** Illustrative distribution of functions between large language models (LLMs) and therapists across a typical psychological therapy workflow.

Therapy phase	Therapeutic function or decision point	Potential LLM contribution	Proposed therapist-delivered contribution
Assessment	Gathering and organizing clinical information	Elicit symptoms, history, goals, and preferences; summarize information; identify missing questions; and organize responses against loaded diagnostic or formulation templates	Establish what information is clinically important; address ambiguity or contradiction; integrate verbal, nonverbal, and relational information; and judge when the available account is unreliable
Formulation	Developing and revising a psychological formulation	Generate candidate rule-based formulations, identify possible maintaining factors, and connect reported difficulties with established psychological models	Develop the formulation collaboratively; incorporate what emerges within the therapeutic relationship; revise the formulation as the person, context, or treatment response change; and integrate nomothetic and idiographic levels of explanation
Treatment planning	Psychoeducation and treatment rationale	Explain symptoms, psychological models, and treatment rationales; adapt language; and adapt examples to stated needs, preferences, and literacy	Determine which explanation is relevant and how it should be framed and adapt in the presence of stigma, shame, confusion, distress, or reassurance seeking
Treatment delivery and structure	Structured therapeutic techniques	Guide established exercises; generate examples, worksheets, and practice tasks; and provide prompts and feedback aligned with an evidence-based protocol	Determine whether the intervention fits the formulation, preference, and present context; model its use where required; and adapt, defer, or discontinue it in cases in which routine delivery is ineffective, poorly tolerated, or unsafe
Active therapy	Therapeutic challenge and responding to avoidance	Identify disengagement and generate alternative perspectives or tasks if prompted	Distinguish fear-based avoidance from realistic danger; identify subtle avoidance, limited capacity, ambivalence, or another barrier; judge whether challenge is indicated; and calibrate its timing, form, and intensity
Active therapy	Using the relationship as a mechanism of change	Generate empathic, validating, and collaborative-sounding language; recall earlier conversations; and maintain a coherent and supportive conversational style	Use the relationship itself as a source of information and change, identify interpersonal patterns as they occur, understand the therapist’s own response as potential clinical information, and support the person to experience and practice different ways of relating in or between sessions
Active therapy	Recognizing and repairing alliance strain	Detect disagreement, disengagement, or negative sentiment and suggest a possible repair response	Recognize subtle withdrawal or passive and active confrontation, determine whether a clinically meaningful rupture has occurred, explore its meaning, acknowledge the therapist’s contribution, negotiate how to proceed, and judge manner of follow-up after dropout
Ongoing treatment adaptation	Calibrating pace, arousal, and level of challenge	Adjust language complexity, task difficulty, or session structure using reported symptoms, preferences, and engagement data	Integrate verbal, nonverbal, relational, and contextual cues to judge the person’s current capacity and determine whether treatment should proceed more slowly, intensify, or change direction
Ongoing treatment adaptation	Adapting when treatment burden exceeds capacity	Monitor reported distress, task completion, and engagement; identify reported burden or capacity reduction; and suggest predetermined lower-burden alternatives	Determine whether the cognitive, emotional, interpersonal, or practical demands of treatment exceed current capacity; reduce, sequence, substitute, or pause elements of treatment accordingly; and provide greater therapeutic or social support to increase capacity
Between-session therapy	Supporting practice and monitoring change	Prompt agreed practice; provide reminders and summaries; review self-reported progress; and identify deterioration, noncompletion, or changes that may warrant attention	Interpret the meaning of noncompletion, deterioration, or disengagement; distinguish ordinary fluctuation from treatment burden, avoidance, rupture, or increasing risk; and decide whether the treatment plan or level of support should change
Review and clinical decision-making	Responding to fixed, escalating, or increasingly reality-detached beliefs	Summarize changes in content, conviction, or distress; identify and flag language associated with possible deterioration or risk; and generate candidate assessment questions	Validate distress without automatically affirming the belief; assess conviction, reality testing, function, and risk; judge whether exploration or challenge is appropriate; and decide whether monitoring, consultation, or escalation is required
Review, escalation, and transition after treatment and discharge	Clinical judgment when current therapy may be insufficient	Summarize the available evidence; identify and flag possible risk or nonresponse; generate preloaded options for alternative treatment, human consultation, or referral options; and support documentation	Judge the limits of the available information and current treatment; seek further evidence; decide whether to continue, adapt, consult, or escalate; discuss the decision with the person; maintain continuity until responsibility is appropriately transferred; and liaise with referrers and other health professionals regarding progress and the ongoing plan

## Operationalizing and Testing the Proposed RAA Functions

The DPR model by Bennett-Levy [10] highlights measurement limitations, including the challenge of evaluating externally observed behaviors and the internal cognitive processes of the therapist. In contrast, declarative knowledge and structured procedural skills lend themselves to direct assessment, which can be conducted through knowledge tests, evidence-based clinical vignettes, treatment manual alignment, and observational competence measures such as the Revised Cognitive Therapy Scale [20]. Similar approaches can be used to compare LLM and therapist performance on factual knowledge, formulation, and the delivery of highly structured psychological interventions. These methods, however, are less suited to the relational function.

The RAA functions require evaluation methods that capture performance in the context of dynamic clinical interactions. Independent coding of live or recorded sessions, responses to standardized video scenarios, and client ratings of the alliance and their session experience and supervision scales are commonly used to quantify these skills. Measures such as the Facilitative Interpersonal Skills task [21], the Working Alliance Inventory [22], and the Session Rating Scale [23] are examples. Automated analysis using AI could eventually make such assessments more feasible.

Research could compare LLM-only, therapist-only, and therapist-plus-LLM responses to standardized “multi-turn” clinical scenarios or, where feasible, recorded therapy session segments. Prespecified and coded behavioral indicators could be developed for each of the functions. Blind expert ratings, client-reported session experience or alliance measures, treatment fidelity, safety indicators, and clinical outcomes could then test whether these functions can be reliably identified and, ultimately, whether they add value beyond structured LLM performance.

## Implications for Workforce Training and Development

Accepting this emerging division of labor shifts the question from whether AI will be involved in therapy to how current and future therapists should be prepared to work alongside it. Current clinicians will need to upskill in the critical, safe, and effective use of AI tools, whereas the next generation will need to be trained from the outset to work in cohesive partnership with these systems without losing the judgment, relationship work, and accountability that remain central to care.

This framework could guide how therapist competence is understood and taught. The DPR model by Bennett-Levy [10] is useful here because it distinguishes between conceptual knowledge, technical skill, and the reflective capacities through which therapists refine their work over time. AI may increasingly support aspects of declarative and procedural work, particularly where tasks are structured, repeatable, and language dominant. Psychologists and therapists of the future may spend less time on generic information delivery, routine between-session prompting, and first-pass documentation.

In contrast, the therapist functions identified in our RAA framework depend more heavily on reflective, perceptual, and contextual expertise. This will allow therapists to spend more time on contextual judgment, therapeutic challenge, care integration, rupture repair, complexity management, and responsibility-bearing decision-making. This shift should influence who is trained, what they are trained to do, and how competence is assessed. If valid, AI may not simply reduce therapist workload; it may change the core focus of therapist initial skill training and ongoing professional development.

There are several implications to consider in this new paradigm. First, training programs may need to place greater emphasis on clinical skills that are often left implicit in training: noticing process, detecting incongruence, judging timing, using the self in therapy, managing relational strain, and adapting under uncertainty. As AI increasingly takes on repetitive and language-dominant tasks, these higher-order interpersonal and judgment-based functions become more central to human clinical value, especially in complex, high-stakes presentations. These capacities are least likely to be acquired through didactic knowledge alone and most likely to require repeated reflective practice, supervision, and experience.

Second, digital literacy will need to become a core clinical competency rather than an optional extra. Clinicians will need to know what to defer to AI tools; what not to defer; how to evaluate bias and safety; how to recognize when AI output is mistimed, context blind, or clinically unsafe; and how to communicate transparently with clients about the role of AI in their care [18]. Tasks can be delegated to AI, but clinical responsibility cannot.

Third, supervision will need to evolve. Supervisors may increasingly need to help trainees not only formulate and reflect on process but also critically review how AI-supported outputs are being used in assessment, planning, communication, and treatment delivery. This includes teaching clinicians how to recognize overreliance, automation bias, erosion of clinical judgment, and the temptation to equate polished output with clinically grounded care.

Fourth, service models may need to become more deliberately stratified [24]. Some clients, depending on their clinical presentations and support needs, may benefit substantially from AI-enhanced or partly automated pathways, particularly where needs are lower intensity, structured, or educational. Others will require more intensive human involvement because treatment depends on alliance, challenge, safety monitoring, trauma-informed pacing, or complex integration across systems. Workforce planning should therefore assume a distribution of roles across a continuum of care intensity, not a single model of “AI therapy.” This also implies different training requirements across clinical care settings rather than assuming a single set of therapist competencies for all AI-assisted practice.

Fifth, this shift raises a challenge for the profession itself. Many of the capacities outlined above are sophisticated, not every therapist demonstrates them consistently, and some clients may not need them. However, if AI absorbs more of the structured and repeatable aspects of care, the comparative value of human therapists may increasingly lie in the parts of practice that are hardest to standardize and most important when stakes are high. At the same time, the future should not be framed only in terms of what AI cannot do. A more useful question is how therapists and AI can work in synergy, with AI extending reach and reducing burden while therapists concentrate more fully on the relational and judgment-intensive parts of care.

Finally, some clients prefer minimal or no AI involvement, which means that therapists will still need strong training in all aspects of traditional face-to-face therapy rather than assuming that all future practice will become AI mediated.

## Conclusion

The key question in the age of LLMs is no longer whether machines will participate in therapy. The more useful question is what functions will stay with therapists as therapy becomes more relationally demanding or more complex. Our clinical perspective is that the enduring therapist role will center on functions that are safety critical, relationship dependent, difficult to specify in algorithmic terms, and not easily automated in a meaningful way. AI may transform therapy and extend its reach, but in doing so, it may also force the field to articulate, with greater precision than ever before, what skilled clinicians actually do and what the future mental health workforce must be trained to protect.

## Abbreviations

DPR: declarative-procedural-reflective
LLM: large language model
RAA: relational, adaptive, and accountability
